# Participant experiences with a text message and contingency management intervention for alcohol use during pregnancy and lactation in Cape Town, South Africa

**DOI:** 10.1186/s13722-025-00594-7

**Published:** 2025-07-29

**Authors:** Lesley-Ann Erasmus-Claassen, Noluthando Mpisane, Petal Petersen Williams, Felicia A. Browne, Bronwyn Myers, Wendee M. Wechsberg, Charles D. H. Parry, Shantae N. Taylor, Yukiko Washio

**Affiliations:** 1 Mental Health, Alcohol, Substance Use and Tobacco Research Unit, South African Medical Research Council, Francie Van Zijl Drive, Parowvalley, Cape Town, 7505 South Africa; 2 Department of Global Health, Institute for Life Course Health Research, Stellenbosch University, Stellenbosch, South Africa; 3 Mental Health and Addiction Research Group, Department of Health Sciences, University of York, York, UK; 4 Substance Use, Gender and Applied Research, RTI International, Durham, NC 27709 USA; 5 Gillings School of Global Public Health, University of North Carolina, Chapel Hill, NC USA; 6 Department of Psychiatry and Mental Health, University of Cape Town, Cape Town, South Africa; 7 National Drug Research Institute Curtin enAble Institute, Faculty of Health Sciences, Curtin University, Bentley, WA Australia; 8 Department of Psychiatry, Stellenbosch University, Stellenbosch, South Africa; 9 Department of Obstetrics, Gynecology and Reproductive Sciences, Temple University Lewis Katz School of Medicine, Philadelphia, PA 19140 USA

**Keywords:** Prenatal alcohol use, Fetal alcohol spectrum disorders, Contingency management, Text messaging, Maternal health

## Abstract

**Background:**

The Western Cape region of South Africa has one of the highest global rates of Fetal Alcohol Spectrum Disorder (FASD), underscoring the urgent need for effective interventions. This qualitative study, designed as a process evaluation, explores pregnant and lactating participants’ perceptions and experiences of a text message and contingency management (CM) intervention.

**Methods:**

The study involved post-intervention interviews with 10 pregnant participants and 10 post-partum lactating participants. Coding and a thematic analysis approach were applied to the collected data using NVivo 12.

**Results:**

Participants identified key factors influencing their engagement in the intervention. Participants faced logistical barriers, but supportive social networks and flexible program components encouraged participation. Increased self-efficacy and external accountability also facilitated behavior change. Furthermore, participants suggested improvements for accessibility and tailored support, highlighting important considerations for future interventions.

**Conclusion:**

The findings highlighted the potential benefits of the intervention in improving individuals’ health behaviors. However, logistical barriers and the need for expanded support services were identified, emphasizing the importance of refining intervention strategies in resource-limited settings.

*Clinical trial registration*: NCT05319977.

**Supplementary Information:**

The online version contains supplementary material available at 10.1186/s13722-025-00594-7.

## Background

Alcohol consumption during pregnancy remains a significant public health concern in South Africa, with national estimates indicating a of maternal alcohol use prevalence of 3.7% [1]. Studies in the Western Cape have reported alcohol consumption rates between 20.2% [2] and 37% [3, 4] among women receiving antenatal care. Prenatal alcohol use is associated with a multitude of adverse effects during pregnancy and birth outcomes including miscarriage, stillbirth, preterm or premature birth, lower infant growth outcomes relating to weight-, height- and head circumference-for-age [5]. Additional risks include intrauterine growth restriction, Fetal Alcohol Spectrum Disorder (FASD) and sudden infant death syndrome [6–9]. FASD refers to a spectrum of neurodevelopmental disorders caused by in-utero alcohol exposure [10]. The reported prevalence of FASD in South Africa is among the highest globally, ranging between 29 and 290 per 1000 live births [11], with the Western Cape region reporting a prevalence ranging from approximately 13.6 to 20.9% (135.1–207.5 per 1000) [12, 13].

Heavy episodic drinking is not limited to pregnancy [1, 14, 15]; it has also been reported during the breastfeeding period [16–18], with one study finding that 71% of participants consumed alcohol during the postpartum period [19]. Alcohol use while breastfeeding can negatively affect breastfeeding duration, lactational performance, breast milk production, and infant neurocognitive development [16, 20]. Studies have shown that post-partum drinking and breastfeeding can lead to children having significantly lower than average height and weight compared to peers not exposed to alcohol [19], lower verbal IQ scores and more anomalies than children whose mothers abstained from alcohol [21].

Comprehensive strategies that integrate education, health promotion, and evidence-based approaches, are crucial for fostering and supporting positive changes in alcohol-related behaviors [22, 23]. Contingency management (CM) is regarded as an evidence-based approach used to address alcohol, tobacco and other substance use-related behaviors by rewarding individuals with incentives such as money, prizes, or vouchers, for positive changes in their health behavior [24–29]. In addition to CM, text messaging and other mobile health (mHealth) initiatives have been used in other low- and middle-income countries to improve appointment attendance [30] improve adherence to treatment regimens, educate patients and promote behavior change [31].

The Women’s Health CoOp (WHC) is a brief intervention designed to address substance use and related issues among women, has shown to be effective in disadvantaged South African populations. Research indicates that using health-promoting messages and text message referrals from the WHC significantly enhances self-efficacy for behavior change [32, 33]. However, the integration of these components with a contingency management (CM) approach remains underexplored, which was the focus of the Maternal Alcohol Reduction Intervention in South Africa (MARISA) study.

The main MaRISA study found promising reductions in alcohol use, with the number of drinking days decreasing and a significant increase in alcohol-negative urine tests. Although reductions in alcohol use during breastfeeding among post-partum participants were observed, these changes were not statistically significant [34]. Notably, depressive symptoms and emotional abuse from a main partner significantly declined over time, suggesting broader psychosocial benefits. These findings support the feasibility of the intervention and the need for further evaluation in a larger, powered trial. This qualitative study was designed as a process evaluation to complement and provide contextual insight into the findings of the main intervention study. Specifically, it aimed to explore participant’s perceptions of and experiences with the combined text messaging and CM intervention for hazardous alcohol use during pregnancy and lactation. The interviews focused implementation, acceptability and participant engagement with the intervention components.

## Methods

We conducted a qualitative study nested within the MARISA study to explore participants’ perceptions and experiences of a text messaging and CM intervention for reducing hazardous alcohol use during pregnancy and lactation. The main MaRISA pilot trial involved 60 participants (31 pregnant and 29 post-partum and breastfeeding at enrolment) and included twice weekly alcohol use monitoring via urinalysis, CM incentives linked to alcohol abstinence, and weekly health promoting text messages. Full details of the trial procedures and outcomes are reported elsewhere [34, 35]. Eligible participants for the intervention were required to have access to a personal mobile phone to receive the weekly text messages (see Supplementary File 1). Previous studies have confirmed high mobile ownership among women in this population [36, 37]. Semi-structured qualitative interviews were conducted with a sub-sample of participants following the 3-month intervention period. These interviews explored key themes, including (1) barriers to participating in the intervention, (2) the facilitators of participating in the intervention, (3) the mechanisms of behavior change, and (4) suggestions for improving the intervention. This qualitative study is presented in line with the COnsolidated criteria for the REporting Qualitative research (COREQ) guidance [38].

### Participant recruitment

Twenty out of a sample of 60 participants, 10 who were pregnant and 10 who were post-partum and breastfeeding at the time of main study enrollment, were randomly selected from the main sample based on a computer-generated random selection of numbers to be interviewed after completing participation within the main study.

All the selected participants were enrolled in the main study and received the pilot intervention. From the random selection, two women who were selected had to be replaced because one withdrew from the main study and the other could not be found during tracking efforts. Both of these selected participants were replaced through another random selection. All of these potential participants consented to a one-hour interview during their original study participation. Interviews were conducted in a private room at the two identified healthcare facilities or in the participant’s home. The interviews were completed with only the participants and the interviewer present.

The interview guide questions were aimed at exploring the participants’ general experience of being a participant in the study, their perceived behavioral adaptations (if any), and their views on the intervention components i.e. CM through financial incentives and receiving weekly health-promoting text messages in short messaging service (SMS) format. Participants were asked to share their perceptions of the content of the text messages and whether the messages impacted their general health behaviors. Participants’ satisfaction with the intervention, its impact, areas of improvement, and recommendations relating to improving future intervention programs were also examined.

### Procedures

Interviews were conducted by the first author (LEC), a native of the Western Cape, who has a post-graduate degree in psychology, is bilingual and has extensive qualitative research experience. At the time of data collection, she fulfilled the role of Project Coordinator on the MaRISA study and had no prior relationship with any of the participants. The participants were asked to provide written informed consent prior to commencing the interviews. Interviews were conducted in person from October 2022 until March 2023. Interviews lasted up to 30 min and were audio recorded and translated while being transcribed. Since the interviews were conducted in both English and Afrikaans, audio recordings of the interviews in Afrikaans were transcribed and concurrently translated into English.

The established semi-structured interview guide was developed to focus discussion points during the post-intervention key informant interviews, but participants’ responses determined further exploration and other discussion points. Participants were reimbursed for their time at ZAR150 (~ USD7.95 [ZAR18.86/USD1]) per participant.

### Data analysis

Reflexive thematic analysis (TA) was used to analyze the data [39, 40] in the format of English transcriptions. According to Braun and Clarke [36], coding reliability approaches prioritize early theme development and consider coding as identifying evidence for predetermined themes, whereas reflexive approaches such as TA, involve later theme development from codes, emphasizing shared meaning emerging from the researcher's interpretative effort. Themes are constructed rather than inherent, and the coding process is organic and subjective, requiring critical reflection from the researcher. In this context a hybrid approach to coding was used, with a deductive approach used to code key concepts asked about in the interview guide and an inductive approach that allowed new codes to emerge from the data. The approach involved six recursive phases: (1) data familiarization and taking notes;(2) data coding; (3) generating initial themes from codes and collated data; (4) reviewing and developing themes; refining, defining and naming themes; and, (6) writing the results in the final phase [39].

NVivo 2020 software was utilized to store and manage the data as well as facilitate coding. This approach also included recording memos, throughout the data collection and analysis process to record thoughts, notes, and observations. The first author (LEC) conducted the initial process of familiarization by reviewing the transcripts. Both the first author and the second author, NM, discussed the initial code definitions and coded the first two transcripts independently. Hereafter, the authors reconvened to discuss the refinement of the themes and the emergent sub-themes, as well as sharing notes reflecting on their personal positionality and understanding of definitions. Combining codes into overarching themes and redefining themes formed part of this process, and once consensus was reached, both coders continued to code the remaining transcripts independently. Any coding disagreements were resolved through discussion and consultation with one of the authors (PPW).

## Results

Participants ranged in age from 19 to 43 years, with a mean age of 28 (SD = 2.12); fourteen (70%) women self-identified as Colored (of mixed-race ancestry), and 6 (30%) women self-identified as Black African. Approximately 95% of the sample reported 12 years or less of education and 65% of participants were unemployed at the time of the interview. The majority of participants (75%) indicated that they had a main relationship partner at the time of the interview.

Participants highlighted various factors that affected their participation and engagement in the intervention, such as logistical barriers and stigma as obstacles (barriers to participation), while supportive social networks and flexible program components encouraged participation (facilitators of participation). Participants also acknowledged that increased self-efficacy and external accountability played a role in their behavior change (mechanisms of behavior change). Furthermore, participants offered suggestions for enhancing accessibility and customising support structures to meet participant needs, highlighting important factors for future interventions.

### Barriers to participating in the intervention

#### Impact of trauma

The intervention program was not easy for all the participants to commit to and follow because of recent traumatic experiences, geographical access to the healthcare facilities and other similar barriers. For one of the participants, it was particularly difficult to follow the intervention program since she had lost her home in a fire. However, she described still having a positive experience due to the tenacity of the field staff in their tracking efforts to find her: “During that time, I felt very different, and that time was the most difficult for me, that the place burnt down. And I was with my aunt for a short time. But they kept on looking until they found me. They left messages and so, but they took the trouble to reach me.” (Participant 2, 38 years old).

#### Influence of structural barriers

Some participants, especially pregnant participants, expressed that they did not like traveling to the health care facilities for their twice weekly alcohol monitoring (intervention) appointments since the activity tired them out: “It was the travelling … We live far so we had to travel and come to the clinic almost two times in a week so I would get tired quickly. That is the one thing that made me get tired of having to keep coming to the program” (Participant 17, 25 years old). However, the travel assistance offered to address geographical barriers was also perceived as an additional incentive by some participants: “…that transport money also helped a lot. That ten rands helped a lot…and actually that was really great, because if it was winter, here, the taxi would be just right here.” (Participant 9, 36 years old).

Some participants reportedly experienced logistical difficulties in receiving the health-promoting text message component of the intervention, i.e. not having access to a mobile phone after enrollment, due to loss or theft, and frequently changing in phone numbers during participation.

### Facilitators of participating in the intervention

#### Positive experiences

Participants expressed that the intervention program provided them with a sense of control over their lives and their drinking habits, as Participant 11 (23 years old) explains: “Oh, the program, it was making me control myself, because I got friends who are drinking and they gonna say, let’s go drink, so I know that by Tuesday, sister is gonna come to me … So, by that time I know I got something that I will be doing so I have to be sure of that”.

The positive view participants had of the research field staff had a profound impact on their daily lives. This could have served as a facilitator in attending the program and influenced the high retention rates. Participants perceived the field staff as friendly and felt cared for, expressing that they had established a good rapport with them. As expressed by Participants’ 6, 9 and 13: “… they were very friendly. I did enjoy coming here to them. To see them and so” (Participant 6, 23 years old). “The staff, because we found a bond, we were like: “Hi, how are you” and so on. So, I will say that the staff was very nice, very friendly and made us feel welcome and so on” (Participant 9, 36 years old). Participant 13 (43 years old) “… only their friendliness, in the morning when they come hoot at you, even if you are not washed, they are prepared to wait for you or we meet you there at the clinic. And it wasn’t for me about the money, it was for me just about the communication and when I get into that van or the vehicle and then my family was cheering for me … even if it’s not Tuesday or Friday, they call you. Are you fine? Are you okay?… Checking in …”.

Additionally, participants valued the transparency of field staff, and the time spent on explaining concepts and procedures prior to an expected activity, as Participant 1 (33 years old) shared “It was so organized. The way they communicate with a person is very nice and they don't make you feel uncomfortable…”. The content of the health-promoting themed text messages also served as a source of motivation, which helped participants gain a sense of self and improve their mental health: “I thought a lot about that SMSs because sometimes when I felt depressed then I read through it again and then it motivates me a bit” (Participant 4).

### Mechanism of behavior change

#### Monitoring alcohol use

Participants shared varied experiences attending alcohol monitoring appointments at healthcare facilities but mainly acknowledged the positive impact of monitoring on reducing alcohol consumption. Regular alcohol monitoring helped participants to reduce their alcohol consumption because they knew they were going to be tested for alcohol use, as Participant 16, 19 years old, explained:”It wasn’t difficult, … because fortunately I don’t live too far, I live near to the clinic, so it wasn’t difficult for me. It actually helped me a lot because I use to be a strong drinker and I came down from it quite a lot and it made me not drink, when I knew I had to be there at that time, because I knew I had to come and give my urine.”. Unfortunately, there were participants who also reported that they struggled to reduce their alcohol consumption and that the weekly alcohol testing was a disincentive to attending the healthcare facility as they were concerned that their alcohol use would be detected. As shared by Participant 13, 43 years old: “To attend the program by using alcohol was very difficult for me, to be honest, it was very difficult, sometimes I didn’t want to come here …. Yes, it was tough … a little tough, yes … I am working as well … very difficult for me, yes.”

In contrast, there were participants who reported that the MARISA intervention helped them make changes to their alcohol and tobacco use, and to their general health, habits and behavior. For example, Participant 4, admitted to heavy drinking during her pregnancy, however, once she entered the program, she was able to abstain from alcohol and permanently quit smoking. Participants explained how these changes in their alcohol consumption improved their living conditions and relationships with their families. They noted that during their enrollment in the program, they refrained from drinking on weekends and were able to dedicate more time to their families. Some participants thought that these changes contributed to a healthier pregnancy. Participant 1, 33 years old, stated that she had “left the alcohol at the end of the day, and I have a healthy baby today.” Participant 13, 43 years old, shared a similar sentiment that attending the program was in her best interest. It helped her to drink less, especially now that she has obtained full-time employment: “At the end of the day, no I tried, when I got deeper into the program, then I am getting a sense of it’s for my best, uhm so I have to attend. So, for me, it’s known I am drinking less and especially now I am working full-time. So, I am drinking less and weekends, I am going to work so there’s no time.”

#### Impact of financial incentives and health promoting text messages

Financial incentives under CM were highly valued and health promoting textmessages, delivered through short messaging services (SMSs), served as a motivational tool for behavior change, instilling a sense of pride and purpose. Participants perceived the content of the text messages to be helpful and expanded their knowledge base: “… it made me realize that the wine goes into the breastmilk and at the end of the day, the baby will drink from my breast. So, yes it did make me think a lot” (Participant 16, 19 years old). In addition, Participant 11 (23 years old), shared that the text messages made her feel proud of herself and further encouraged her to motivate others in a similar situation to join the program.: “Yes, I received SMSs from you … these SMSs were making me to be proud of me, hey, and to know what I have to do for my life. Yeah, from these SMSs they make me to tell others that they are supposed to join in the group if they got a problem like mine, they have to join so they can be alright”.

The CM component seemed to also benefit participants in other spheres of their lives, for instance, Participant 1, 33 years old, used the incentive to support her children while unemployed.: “Like when I got that money … I could go and take out the lay-by. And then my other two kids, I bought each one of them something. So, it helped me a lot because I'm not working at the moment … [was] a big help for me yes.” One participant reduced her alcohol consumption due to the intervention, but her reduction also had a positive impact on her social network with one friend also reducing her alcohol consumption: “My one friend doesn’t drink anymore, even though her child is big … She says it is from you not wanting and buying beer all the time. Because I used to say to her, ‘E’ come let’s buy a box. Yes, I just used to buy a box. Then the two of us combine, then she says come we buy a box. Then we buy a box (referring to boxed wine) … But now…she also stopped … Now, no I don’t want wine. I don’t drink anymore.” (Participant 4, 27 years old). Participants shared that receiving financial rewards for abstaining from alcohol use helped them to stay motivated, as 25 year old Participant 17 shares… “Because it was motivating us a lot to stop drinking alcohol and get some money so that we can buy things that we need to buy for ourselves during the pregnancy” (Participant 17, 25 years old).

Despite their awareness of the risks associated with drinking during pregnancy, participants admitted to consuming alcohol. They acknowledged receiving such information from health education materials and healthcare providers at the facility where they were receiving antenatal care. Many of these participants emphasized the value of the intervention, noting its role in reducing or completely halting their alcohol consumption. “What I learned is just that it is not necessary to use alcohol during your pregnancy, it would be better if you do without it than to have it, you will have to bear the side effects. The poor little one will suffer at the end of the day. You will end up having an alcohol syndrome baby.” (Participant 12, 29 years old).

In addition, participants who were post-partum and breastfeeding reported gaining new insights into the potential impact of alcohol use while breastfeeding, like Participant 18, 23 years old, who shared, ‘When you breastfeed, when you drink alcohol and breastfeeding, you are giving the baby also the alcohol, so …it’s not healthy for the baby.” Whereas another participant shared how her perception had evolved while participating in the program… “I knew now that it was dangerous to drink when you breastfeed, not that I did not know it, but it gave me a bit more insight” (Participant 9, 36 years old).

### Suggestions for improving the intervention

#### Alternative rewards

Participants expressed their interest in alternative types of rewards such as vouchers from local supermarkets or retailers. As Participant 20 (22 years old) stated: “you can sometimes add vouchers, hey. And things like that, maybe for the babies, vouchers, for the things when the person is pregnant, they get cravings right, maybe a voucher or something, yeah.”

#### Family planning

Other suggestions to improve the intervention program included education on family planning to prevent unplanned and/or unwanted pregnancies and testing for other substances in the program since women were only monitored for alcohol use with an ethyl glucuronide (EtG) test: “You can educate on substances like drugs and so … not only alcohol, but preventing pregnancy …” (Participant 20, 22 years old) and “For me maybe, a drug testing … I would like a drug test to be added, then it would be better for me.” (Participant 4, 27 years old).

#### Counselling

Expanding the intervention components to include behavior change counseling and incorporating recreational activities to address boredom were some of the other suggestions that participants had to improve the received program. “You need to sit with them and counsel them. To show them how it destroys them, help them … And do activities … Just to keep, uhm the people busy …” (Participant 18, 23 years old), and “They need counselling. You can put more information about … that drugs are not right for your health and then you can use, you can actually put more information in and try to help them … To not walk around … not think about that, that I have to smoke now.” (Participant 19, 21 years old).

#### Support groups

The need for support groups incorporated into intervention programs was raised by the majority of participants, even though participants were demographically from different communities within the Western Cape: “Here are no support groups, nothing, there’s nothing. Look here in [my community], there’s no life, there isn’t even any work, no income. It is needed because here is nothing.” (Participant 10, 36 years old).

#### Inclusion of partners and addressing intimate partner violence (IPV)

Participants recommended that future intervention programs should include main partners and focus on strategies to prevent intimate partner violence (IPV), teach men better coping mechanisms other than using violence and focus on educating men on gender roles, as one participant elaborates about sharing responsibilities within a relationship: “With my partner then there must be a program for the abusive partner. To maybe say the man must do that, is all that the woman wants. You can’t expect from your side alone and then you don’t do your part … [speak about] Gender roles … One hand helps the other hand…” (Participant 2, 38 years old).

Similarly, they thought support groups can explore the positive characteristics of being a supportive partner: “Maybe not to abuse your woman. Or sleep around. Cheating and such stuff. Or to be there for your child. Even if you're not with the mother but support your child. Because some fathers, they want to be in the child's life. But then they get avoid [ignored] by the mum or she's getting avoided [ignored] by the other mother (grandmother). And then you get that father that just don't care.” (Participant 6, 23 years old).

## Discussion

The purpose of this study was to explore the perceptions of participants who received the MaRISA intervention. The findings from this process evaluation provide insights into how the intervention was received and implemented, which can inform the refinement and design of similar interventions in the future. The purpose of this study was not to comprehensively explore general barriers to behavior change, but rather to understand how this specific intervention was experienced by participants and the factors that contributed to its effectiveness. Participants reported positive outcomes, including reduced alcohol consumption, improved overall well-being, and positive impacts on their peer network. Notably, participants reported sustained abstinence from alcohol post-intervention, highlighting its potential for long-term benefits. Additionally, supporting literature from various studies affirms the CM approach's effectiveness in curbing excessive alcohol consumption across different demographics [24, 25, 41]. However, some participants cited logistical difficulties, such as transportation issues and travel distance to the healthcare facilities, which made the bi-weekly urine sample submissions for the program cumbersome.

Contingent financial rewards from the MaRISA intervention for abstaining from alcohol supported participants in meeting family needs and starting business ventures, leading to health improvements, behavior changes, and long-term financial security. The success of such monetary and suggested non-monetary incentives in this and other studies [42, 43] underlines the effectiveness in encouraging sustained recovery and healthier choices during key life stages [44, 45].

Participants appreciated receiving weekly texts about the risks of drinking alcohol during pregnancy and while breastfeeding, finding the messages educational and motivational. Similarly, a study conducted by Lau and colleagues [51], among pregnant women attending a primary health care facility in Cape Town, revealed that text messages containing antenatal health information served as a source of positive reinforcement for the participants and helped to improve health-related behaviors. Although participants who received the health-promoting themed text messages weekly found it useful and motivating, it is important to note that not all the participants received the text messages. Some, however, were missed due to a lack of mobile access or changing phone numbers frequently. Mekonnen et al. [52] speak to this, having found that low mobile ownership is a barrier that could have an impact on mobile health intervention implementation. Addressing these barriers are crucial for the success of such digital interventions in resource-limited and low socioeconomic settings [47, 48]. Despite these challenges, overall findings indicate that text messaging shows promise in promoting healthier behaviors among pregnant and lactating individuals.

To enhance the program, participants suggested adding counseling for behavioral change, education on family planning [49], testing for other substances, and incorporating other recreational activities [37, 45]. There was a call for a focus on intimate partner violence and gender roles, linking alcohol use to IPV [46] and substance use as a way to cope with abuse [23]. Furthermore, individuals are more likely to drink during pregnancy if their partners are not good sources of support [46]. These user recommendations are backed by research [4, 15], which emphasizes the importance and effectiveness of health education [4, 15, 37, 47–50], as well as the impact that incentive-based interventions for reducing alcohol use can have during pregnancy and lactation [25–28].

It is important to consider the limitations of this study when interpreting its results. This sub-study focused on exploring the perceptions and experiences of 20 participants from only two communities in the Cape Metropole of the Western Cape Province of South Africa. Therefore, the representativeness of findings for individuals who consume alcohol while pregnant or post-partum and who are breastfeeding in South Africa remains limited.

## Conclusion

This sub-study provides valuable insights into how a textmessaging and CM program could aid South African pregnant and postpartum individuals in reducing alcohol use, also positively affecting their peers. Suggestions for program enhancement included integrating behavior change counseling, recreational activities and using a support group format. The findings underscore the value of participant feedback in refining future interventions for alcohol use reduction among pregnant and breastfeeding individuals.

## Supplementary Information

Additional file1 (PDF 3928 KB)

## Data Availability

No datasets were generated or analysed during the current study.
