# Supplementary material for: Participant experiences with a text message and contingency management intervention for alcohol use during pregnancy and lactation in Cape Town, South Africa
Source: Addict Sci Clin Pract. 2025 Jul 29;20:59. doi: 10.1186/s13722-025-00594-7 (PMC12309210; doi:10.1186/s13722-025-00594-7)
Supplement: Supplementary file 1 — Additional file1 (PDF 3928 KB) [file 13722_2025_594_MOESM1_ESM.pdf]

Table 1: Supplementary File 1_MaRISA Text Messaging Intervention - Text messages sent to participants weekly as part of the health promotion text messaging intervention component

TXT DISTRIBUTION	PRENATAL	RESPONSE EXPECTATION
**WK 1 ONLY**	**Congratulations on your decision to stay sober during your pregnancy. Drinking while pregnant can impact your baby's growth.**	
WK 2-WK13(MUST)	"Alert your provider if you feel that your baby stopped moving"	
WK 2-WK13(MUST)	"Alert your provider if you see your vaginal bleeding or smelly discharge"	
WK 2-WK13 (MUST)	"Please ask your provider about family planning options"	
WK 2-WK13 (MUST)	"You can drink or eat gingers to cope with nausea."	
WK2-13	It is good to take folic acid and iron while you are pregnant. Do you want to know which foods have folic acid and iron?	If "YES-send Referrals
WK2-13	Make sure you are eating healthy foods, like greens. Your baby has started to absorb nutrients now.	
WK2-13	You have made a good decision trying to stay sober during your pregnancy. 🤰 We're here to support you to continue doing that.	
WK2-13	It will also be good for your baby if you stay away from smoking.	
WK2-13	Make sure you drink a lot of water during pregnancy	
WK2-13	Tik, dagga, and Mandrax are harmful to babies. It will also be a good thing for your baby if you stay away from these.	
WK2-13	The effect of drinking on a baby may not show up right away. Sometimes it is not seen until they are school age.	
	POSTPARTUM	
**WK 1 ONLY**	**Congratulations on your decision to stay sober during breastfeeding. Drinking while breastfeeding can impact your baby's growth.**	
WK 2-WK13 (MUST)	"if you drink alcohol, breast feed first and then drink equal water to alcohol."	
WK 2-WK13 (MUST)	"Please check the nappies and clean the baby's bottom"	
	**BOTH PRENATAL AND POSTPARTUM**	
WK2-13	(It's the Monday, T, W, Th, F, weekend). Which emoji best represents how you are feeling? (😊, 😳, ☹)	IF, "☹" – send Referrals
WK2-13	". Drinking lots of water can help with pooping. If it does not help, please talk to your provider."	
WK2-13	"It is normal to feel like drinking, smoking, or using drugs again after you decided to stop. Text "YES" for referrals!"	If "YES-send Referrals
WK2-13	"Have you taken care of yourself? Treat yourself with something nice and pleasant."	
WK2-13	It is a good idea not to drink alcohol or use drugs before having sex.	
WK2-13	Do you feel urge to drink alcohol or use drugs when you feel stressed?	YES OR NO; If "YES-send Referrals
WK2-13	Do you have ways to relieve stress other than drinking alcohol or using drugs?	YES OR NO; If "YES-send Referrals
WK2-13	Do you need someone to talk to?	YES OR NO; If "YES-send Referrals
WK2-13	Did you know fermented apple juice has alcohol?	
